# ETS Family Transcription Factors in Gastric Cancer and the Role of ELF3 in the Core Metaplasia Transcription Factor Network

**DOI:** 10.32604/or.2025.069230

**Published:** 2025-11-27

**Authors:** Ioannis A. Voutsadakis

**Affiliations:** 1 Holden Comprehensive Cancer Center, University of Iowa Hospitals and Clinics, Iowa City, IA 52240, USA; 2 University of Iowa Carver College of Medicine, Iowa City, IA 52240, USA; 3 Section of Internal Medicine, Division of Clinical Sciences, Northern Ontario School of Medicine, Sudbury, ON P3E 5J1, Canada

**Keywords:** Stomach adenocarcinoma, transcription factors, gastric, signaling, prognosis

## Abstract

**Background:**

E26 transformation-specific (ETS) family transcription factors have confirmed roles in several types of cancers. This study aimed to clarify the role of ETS family transcription factor alterations in gastric cancers.

**Methods:**

This study examines molecular alterations of ETS transcription factors in gastric adenocarcinomas based on an analysis of publicly available cohorts from the Protein Atlas and the Cancer Genome Atlas. The expression and relationships of members of the ETS transcription factor family with other important factors in the process of gastric carcinogenesis were evaluated using the same resources.

**Results:**

mRNA expression levels of ETS family members in gastric carcinoma tissues were variable, with ELF3, ETS2, EHF, ERF, and ELF1 being the family members with the highest expression. Mutations in individual transcription factors of the ETS family were rare in gastric cancers. The family member ELF3 was well expressed in the mRNA level in a subset of gastric cancers (n = 91), and its expression correlated with the expression of other transcription factors involved in gastric cancer pathogenesis, including HNF4A, HNF1A, CDX2, GATA4, GATA6, and EHF. Cancers with high co-expression of ELF3 and HNF4A were frequently chromosomally instability (CIN), intestinal-type adenocarcinomas, and harbored *TP53* mutations and *WWOX* deletions.

**Conclusion:**

Expression of E74 like ETS transcription factor 3 (ELF3), an ETS transcription family member, correlates with expression of other key factors in gastric cancer and confers specific characteristics that may become exploited in targeted therapeutic interventions.

## 1 Introduction

Gastric cancer is one of the most prevalent and aggressive human cancers [1]. Despite a better understanding of its pathophysiology, prognosis remains grim for patients with metastatic disease, and although a few targeted therapies have been introduced, cytotoxic chemotherapies are the main option for most patients [2]. Gastric cancers have been classified by the Cancer Genome Atlas (TCGA) project into four subtypes, according to their genomic characteristics [3]. The first subtype includes microsatellite instability (MSI) high cancers and possesses mutations or epigenetic alterations in mismatch repair (MMR) proteins MSH2 (MutS Homolog 2), MSH6 (MutS Homolog 6), MLH1 (MutL Homolog 1), or PMS2 (Postmeiotic Segregation increased 2). The chromosomal instability (CIN) high group of gastric cancers displays mutations in the gene encoding for the p53 tumor suppressor and frequent mutations in receptor tyrosine kinase pathways, while Epstein-Barr virus (EBV) associated cancers express the viral product EBV encoded RNA (EBER) and present frequent mutations in PI3K kinase catalytic subunit *PIK3CA*. The last subtype is called genomically stable (GS) and presents frequent E cadherin (*CDH1*) and *RHOA* mutations [3]. A clinical approximation of genomic categories may be obtained through immunohistochemistry in histologic sections for MMR-related proteins, p53, E-cadherin, and *in situ* hybridization for EBER [4].

The E26 transformation-specific (ETS) family of transcription factors has 28 family members divided into 12 sub-families [5]. All members contain an ETS domain (DNA binding domain) and 11 members contain PNT (pointed) domains, which facilitate heterodimerization with other ETS factors, DNA-dependent protein kinases, Androgen Receptor (AR) and AP-1 (Activating Protein 1, a complex of JUN and FOS family transcription factors) [5]. The length of ETS family transcription factors varies between 238 to 663 amino acids, and their core binding sequence on DNA consists of the four-nucleotide GGA(A/T) pattern. The H3 helix in the ETS domain contacts the major groove of the double DNA helix on the target sequence [6]. The 28 genes of the family are located in several different chromosome arms. Outside the sub-families, the family members present limited homology, besides in the ETS domain.

Activation of ETS family members in cancer occurs downstream of the RAS/ERK/MEK cascade and DNA binding sites frequently co-exist with binding sites of the AP-1 dimers, which are also activated by RAS/ERK/MEK [7]. The status of activation of the RAS/ERK/MEK pathway is critical for the output of the co-operative actions of the two families of transcription factors. In addition, both ETS family and AP-1 binding sites co-exist with binding sites of other key transcription factors regulating gastric carcinogenesis, such as GATA4, GATA6 and KLF5 [8]. Two prominent members of the ETS family in the pathogenesis of specific cancers are FLI1 in Ewing sarcoma (translocation EWS-FLI1) and ERG in prostate cancer [9,10]. In gastric cancer, a role of the up-regulation of family member ELF3 has been suggested as part of the network of transcription factors up-regulated by triggers inducing intestinal metaplasia [11,12]. This network may be targeted therapeutically to reverse the carcinogenesis process. This article investigates the role of alterations in ETS family members and their associations with common genomic abnormalities in gastric cancer to provide a better understanding of carcinogenesis mechanisms.

## 2 Methods

### 2.1 Retrieval of mRNA and Protein Expression

Information for the expression of mRNA and proteins of interest in normal gastric epithelium was retrieved from the Human Protein Atlas (https://www.proteinatlas.org/ (accessed on 01 June 2025)) [13]. Levels of mRNA were reported in normalized transcripts per million (nTPM). The protein expressions were reported semi-quantitatively based on the ratio of cases staining positive and the intensity of staining.

The analysis of genomic alterations of the ETS family transcription factors was undertaken in the publicly available gastric cancer cohort of The Cancer Genome Atlas (TCGA; https://www.cBioPortal.org/study/summary?id=stad_tcga_pan_can_atlas_2018 (accessed on 01 June 2025)) [3]. The TCGA gastric cancer cohort encompasses 440 patients and provides publicly available, de-identified patient-level information including clinical and genomic tumor characteristics [3]. TCGA has used a whole-exome next-generation sequencing approach for genomic analyses.

### 2.2 Data Analysis of Genomic Alterations in ETS Family Transcription Factors and of mRNA Expression

TCGA collaboration has generated data on all genomic alterations, including mutations, copy number alterations, structural variants, as well as data on levels of mRNA expression. Diverse calling pipelines were used for single-nucleotide mutation calling by the research institutions that provided genomic data to TCGA [14]. Some patients lacked mutation data for the genes of interest and therefore the analyses were based in a smaller number of patients than the total number in the series. For normalization of mRNA expression from RNA Sequencing (RNA-Seq) data, TCGA used an algorithm called RNA-Seq by Expectation Maximization (RSEM) [15]. This algorithm can handle input data without a requirement for a reference genome. Up-regulation of ELF3 mRNA expression was defined as a normalized mRNA expression *z* score above 0.5 and up-regulation of HNF4A mRNA expression was defined as a normalized mRNA expression *z* score above 1. The selection of the *z*-score cut-off for ELF3 (>0.5) was based on the fact that this cut-off was close to the mean expression *z*-score in the series (0.48). The selection of the *z*-score cut-off for HNF4A was based on previous work showing that, in gastric cancers with induction of the gastric intestinal metaplasia network centered on CDX2 induction and SOX2 suppression, the mean HNF4A expression *z*-score was close to 1 [12,16].

TCGA measured chromosomal instability with a score called aneuploidy score (AS). The score is calculated in each sample by adding the number of chromosome arms that have copy number alterations (gains or losses). The definition of a chromosome arm to be considered copy number altered, gained, or lost, for the AS calculation is to have somatic copy number alterations in more than 80% of its length, as calculated by the ABSOLUTE algorithm from Affymetrix 6.0 SNP arrays [17]. Chromosome arms with somatic copy number alterations in 20% to 80% of the arm length were considered indeterminate. Chromosome arms that had somatic copy number alterations in less than 20% of their length in a sample were considered not altered. A second measure of aneuploidy is provided in TCGA in the form of the Fraction Genome Altered (FGA), which measures the fraction of the genome with copy number gains or losses.

All primary analyses were performed based on data contained in the cBioportal for cancer genomics site (www.cbioportal.org) [18,19]. The group of gastric cancers with high ELF3 mRNA expression, defined as an mRNA expression *z* score relative to normal samples above 0.5 and high HNF4A expression, defined as an mRNA expression z score relative to normal samples above 1, was compared with the group of gastric cancers with low ELF3 mRNA expression, defined as an mRNA expression *z* score relative to normal samples below 0.5 and low HNF4A expression, defined as an mRNA expression *z* score relative to normal samples below 0.5. The two groups were constructed from the TCGA gastric cancer cohort through cBioportal, using the “mRNA expression *z*-scores relative to normal samples” download functionality.

### 2.3 Evaluation of Pathogenic Effects of Mutations

The pathogenic implications of mutations were evaluated using the OncoKB knowledgebase (www.oncokb.org), which is a manually curated publicly available data site providing information on the pathogenicity of genetic alterations of cancer, analyzing available sources [20,21]. OncoKB categorizes mutations encountered in cancer cases as oncogenic, potentially oncogenic or neutral.

### 2.4 Statistical Analysis

The statistical evaluation of significance was performed with Fisher’s exact test or the χ^2^ test for categorical variables, and for continuous variables with the *t*-test. Correlations of expressions of genes of interest were calculated with the Pearson’s correlation coefficient and the Spearman’s rank correlation coefficient, corrected for multiple comparisons. No correction was performed for the limited number of comparisons of gene expression between the two groups according to the level of ELF3 and HNF4A expression. Survival analyses were performed using Kaplan-Meier survival curves analysis. Kaplan-Meier curves were compared with the Log Rank test. All statistical comparisons were considered significant at the level of *p* < 0.05.

## 3 Results

### 3.1 mRNA and Protein Expression

**Figure 1:**
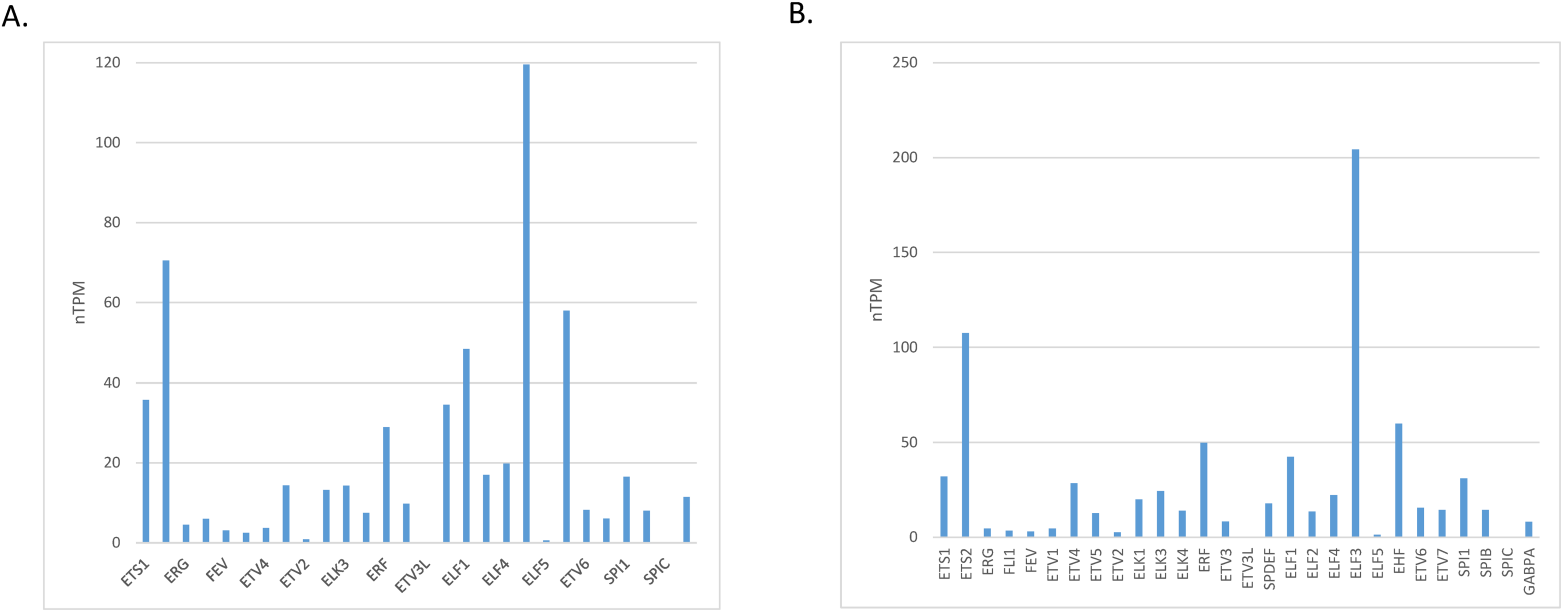
mRNA expression of ETS family members in: (**A**). normal gastric epithelium and (**B**). gastric carcinoma

**Figure 2:**
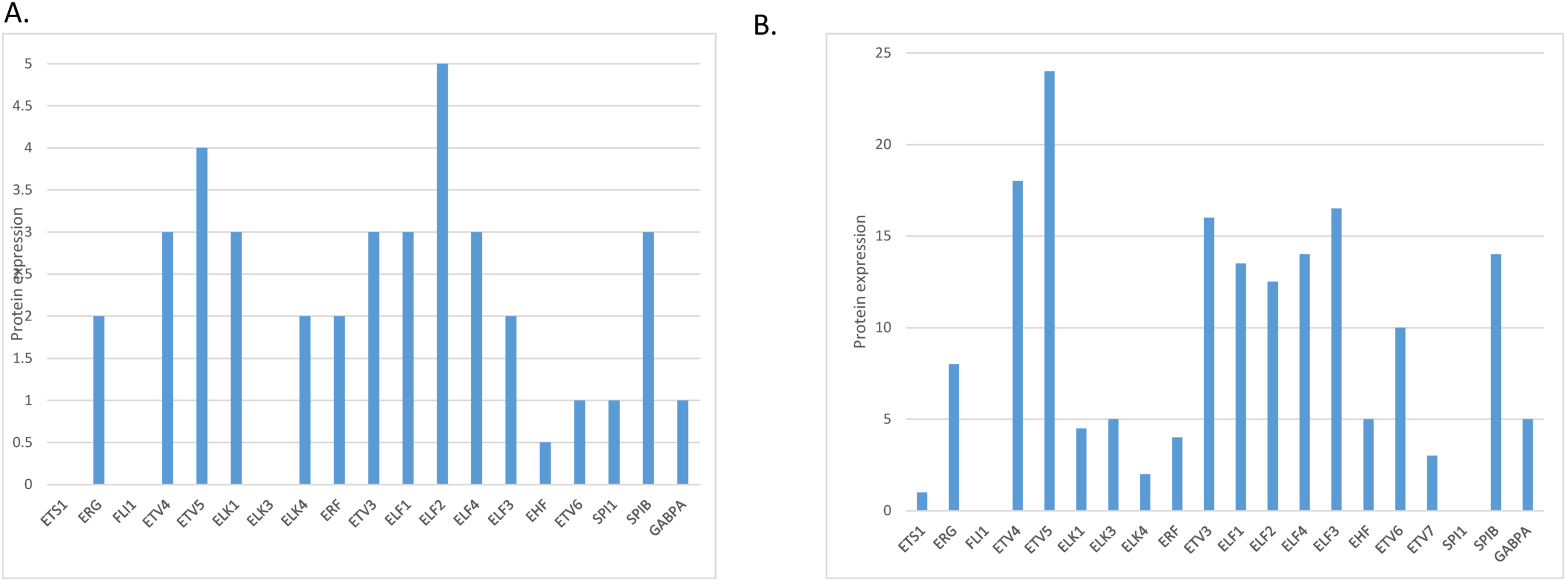
Protein expression of ETS family members in: (**A**). normal gastric epithelium and (**B**). gastric carcinoma

The level of mRNA expression of ETS family members in normal gastric gland tissues varies, with ELF3, ETS2, EHF and ELF1 showing the highest expression (Fig. 1A). At the protein level, members ELF2 and ETV5 showed the highest expression in normal gastric gland tissues and several other family members had intermediate levels, in the Human Protein Atlas (Fig. 2A). The level of mRNA expression of ETS family members in gastric carcinoma tissues was also variable with ELF3, ETS2, EHF, ERF and ELF1 being the family members with the highest expression (Fig. 1B). ETS family members with the highest protein level of expression in many gastric cancer samples include ETV5, ETV4 and ETV3, while ELF2 and ELF3 had moderate to high levels of expression in all samples tested in the Human Protein Atlas but only in one of the two antibodies checked for each protein (Fig. 2B) [13].

### 3.2 Analysis of Genomic Alterations in ETS Family Transcription Factors

**Figure 3:**
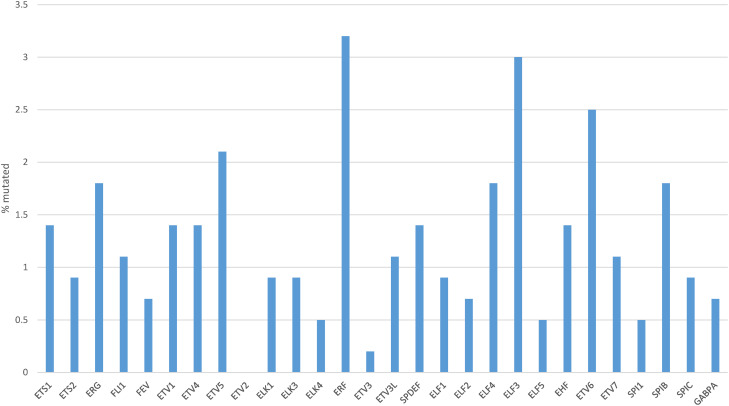
Percentage of mutations in ETS family members in gastric cancer. Data are from The Cancer Genome Atlas (TCGA)

**Figure 4:**
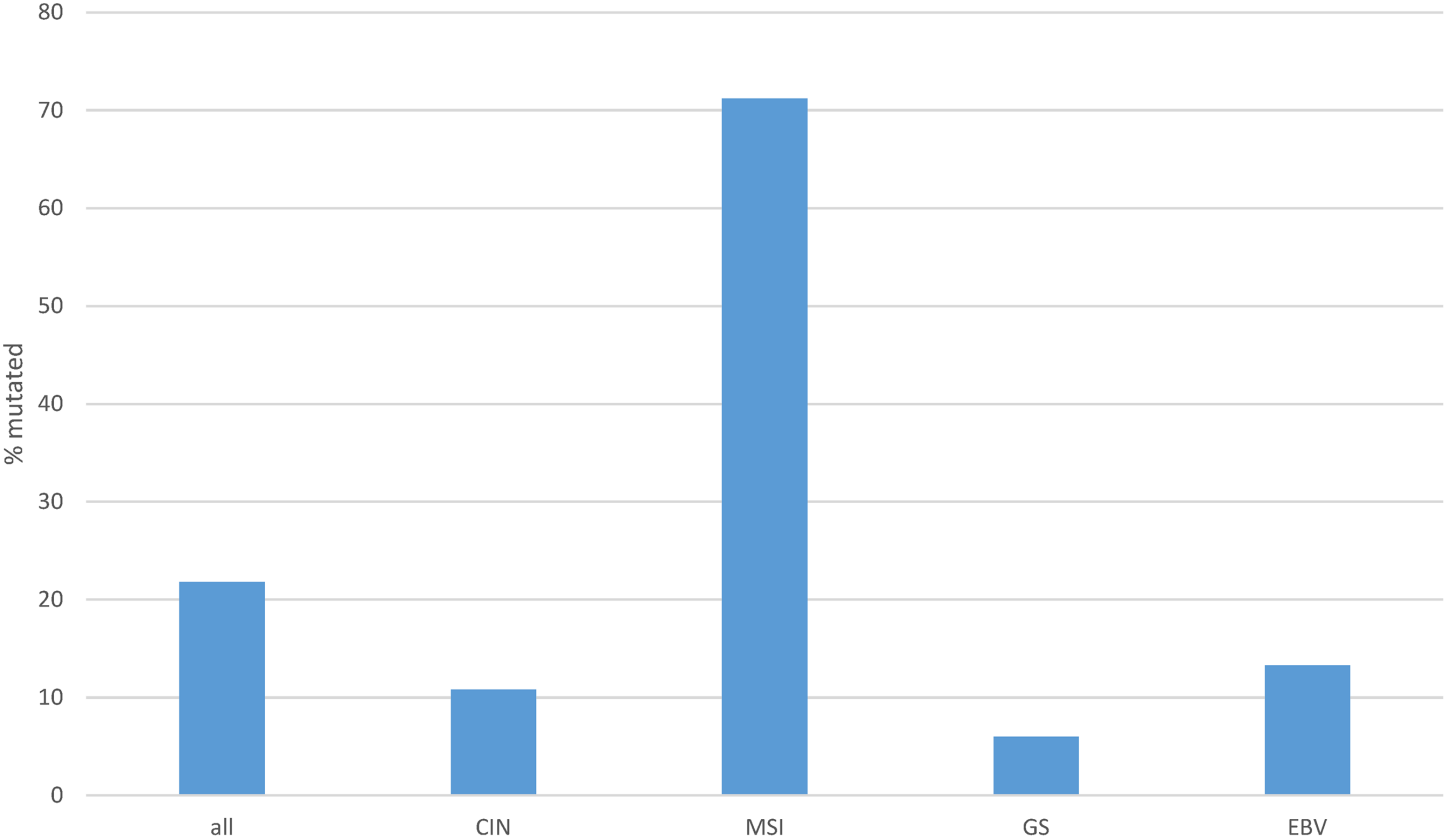
Cumulative percentage of mutations in ETS family members in gastric cancer as a whole and according to genomic sub-types. Data are from The Cancer Genome Atlas (TCGA). CIN, chromosomal instability; MSI, microsatellite instability; GS, genomically stable; EBV, Epstein-Barr virus

The rate of mutations in individual transcription factors of the ETS family was low in gastric cancers. The most prevalent mutations were observed in transcription factors ERF (3.2%), ELF3 (3%), ETV6 (2.5%) and ETV5 (2.1%), with all other members of the family being mutated in 0 to less than 2% of cases (Fig. 3). However, collectively, about one in five gastric cancers (96 of 436 of patients with data available, 22%) bore mutations in one or more ETS family members (Fig. 4). A significantly higher percentage of gastric cancers with high MSI (71.2%) had mutations in ETS family members compared to gastric cancers with high CIN (10.8%), genomically stable (GS) cancers (6%) and EBV-associated cancers (13.3%, Fig. 4). Most ETS family mutations were of unknown significance, while a smaller percentage of mutations were considered pathogenic or potentially pathogenic (MSI high group: 12.3% of the entire MSI high cohort, CIN high group: 0.9%, GS group: 2% and EBV-associated group: none).

**Figure 5:**
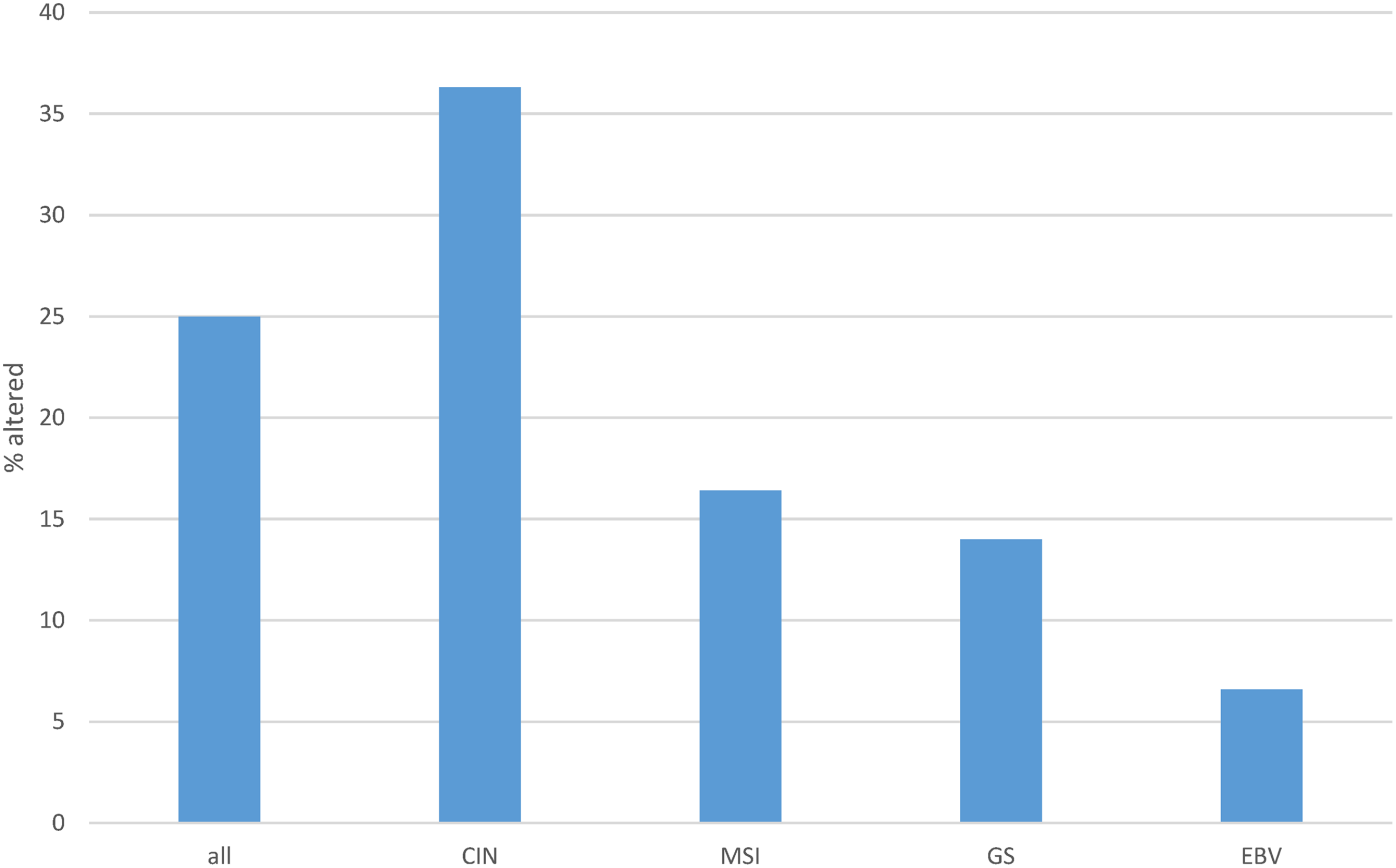
Cumulative percentage of copy number alterations in ETS family members in gastric cancer as a whole and according to genomic sub-types. Data are from The Cancer Genome Atlas (TCGA). CIN, chromosomal instability; MSI, microsatellite instability; GS, genomically stable; EBV, Epstein-Barr virus

**Figure 6:**
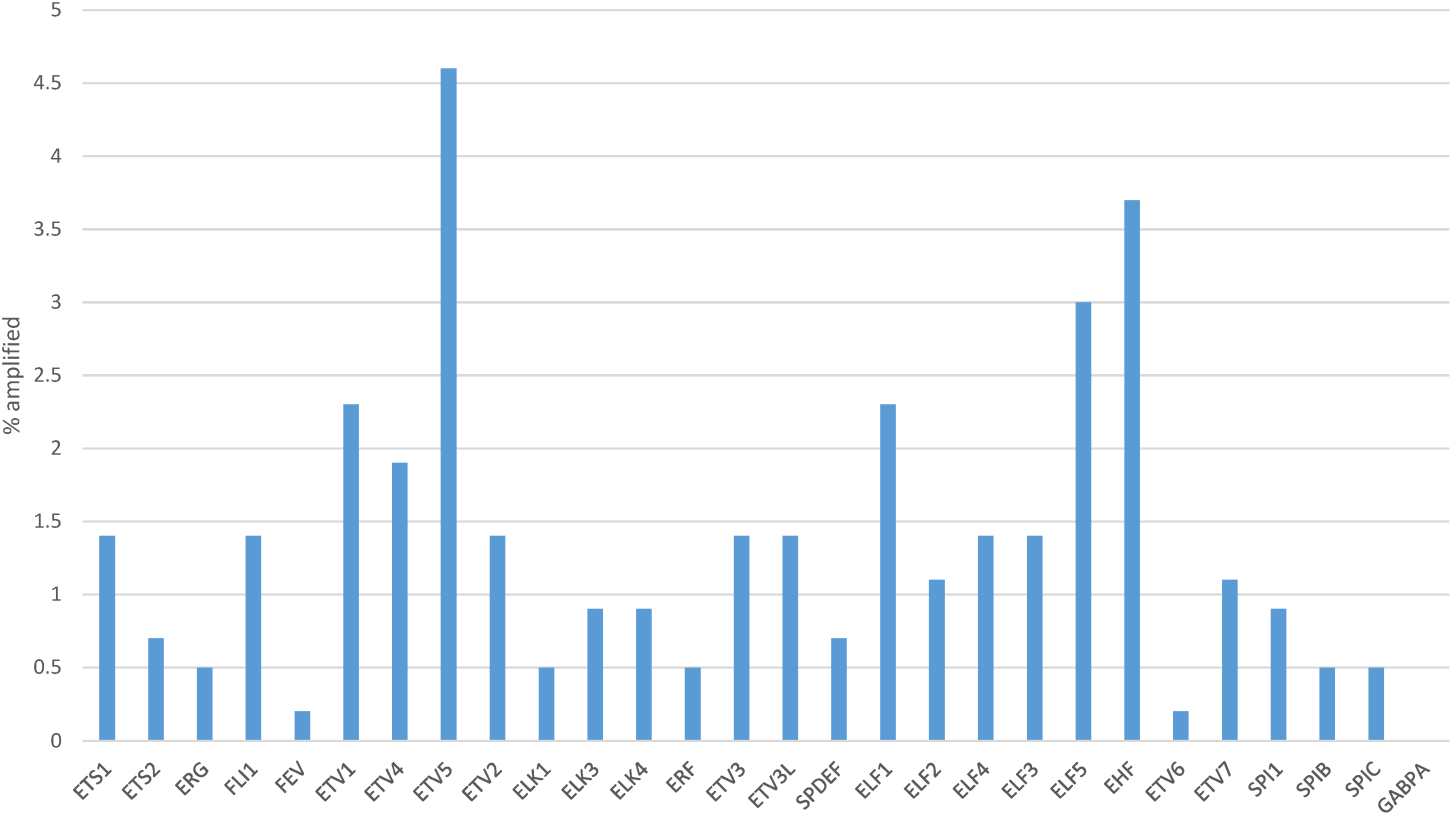
Percentage of copy number alterations in ETS family members in gastric cancer. Data are from The Cancer Genome Atlas (TCGA)

One or more copy number alterations in ETS family genes were present in about one in four gastric cancers (25.8%) of TCGA cohort (Fig. 5). Three transcription factors of the family were amplified in 3% or more of cases (ETV5: 4.6%, EHF: 3.7% and ELF5: 3%, Fig. 6). Copy number alterations were more frequent in the group of gastric cancers with high CIN (36.3%), compared with MSI high cancers (16.4%), GS cancers (14%) and EBV-associated cancers (6.6%, χ^2^ *p* = 0.00001, Fig. 5).

**Figure 7:**
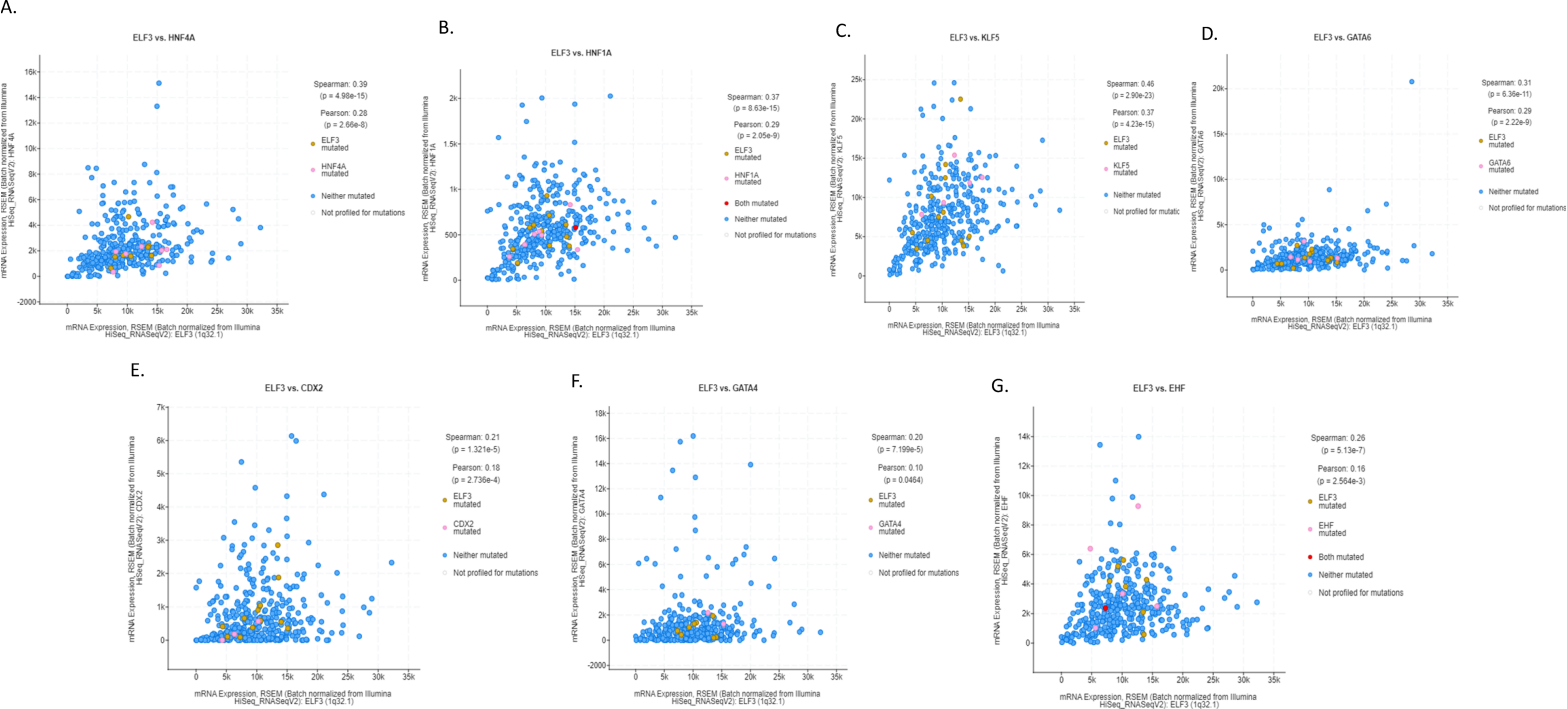
Correlation of mRNA expression of ELF3 with: (**A**). HNF4A, (**B**). HNF1A, (**C**). KLF5, (**D**). GATA6, (**E**). CDX2, (**F**). GATA4, and (**G**). EHF

ELF3 is one of the ETS family transcription factors that was expressed in gastric cancers at higher levels than in normal epithelium and was mutated in a small minority of gastric cancers, representing one of the most prevalently mutated factors of the family (Figs. 1–3). In addition, ELF3 has been described as part of the network of transcription factors induced by intestinal metaplasia and associated with CDX2 and HNF4A induction during the process [11]. Among transcription factors of this network up-regulated in intestinal metaplasia and gastric cancer, ELF3 over-expression is strongly correlated with the over-expression of HNF4A (Spearman’s rank correlation coefficient q = 8.68 × 10^−14^, Fig. 7A), as well as with over-expression of HNF1A (Spearman’s rank correlation coefficient q = 1.42 × 10^−13^, Fig. 7B), KLF5 (Spearman’s rank correlation coefficient q = 4.12 × 10^−21^, Fig. 7C) and GATA6 (Spearman’s rank correlation coefficient q = 5.22 × 10^−10^, Fig. 7D). In addition, strong positive correlation of the expression of ELF3 was observed with CDX2 (Spearman’s rank correlation coefficient q = 4.26 × 10^−5^, Fig. 7E), GATA4 (Spearman’s rank correlation coefficient q = 2.03 × 10^−4^, Fig. 7F), and the other ETS family factor, EHF (Spearman’s rank correlation coefficient q = 2.13 × 10^−6^, Fig. 7G). HNF4A and the related HNF1A factor are the most up-regulated transcription factors of the network in the gastric cancer cohort, with a mean expression (normalized mRNA expression *z* score) of 0.711 and 0.866, respectively (Table 1). CDX2 had the third highest mean expression among these factors and presented also a high percentage of up-regulated cases, as defined by a normalized mRNA expression *z* score >1 (Table 1). In contrast to the network of intestinal metaplasia-associated transcription factors, the upper gastrointestinal tract specification factor, SOX2, was down-regulated.

**Table 1:** Mean expression (normalized mRNA expression *z* score), standard deviation (SD) and percentage of cases with expression of core transcription factors of the ELF3 network having a *z* score above 1 in the gastric cancer cohort of TCGA

Statistic	CDX2	SOX2	HNF4A	HNF1A	GATA4	GATA6	KLF5	ELF3	EHF
Mean	0.636497	−1.24042	0.711174	0.866776	0.133707	0.380942	0.465042	0.484213	0.385338
SD	0.711204	1.763142	0.413546	0.527512	0.675652	0.484946	0.419588	0.332673	0.3557
% *z* score >1	33.42246	9.893048	18.98396	45.9893	5.614973	3.743316	3.475936	1.069519	1.604278

### 3.3 Analysis of Genomic Alterations in Gastric Cancers with ELF3 and HNF4A Over-Expression

Next, a comparison of the sub-set of gastric cancer patients with up-regulated expression of ELF3 and HNF4A with patients who displayed no ELF3 and HNF4A mRNA up-regulation was performed. HNF4A, besides showing a highly significant co-expression with ELF3, has been identified in a study of master transcription factors of gastrointestinal cancers to be a target gene of lineage-defining master transcription factors [11]. These factors coordinately promote the up-regulation of HNF1A and the interleukin inflammatory pathway and cell proliferation. The group with ELF3 and HNF4A mRNA up-regulations included 49 patients and the group with no up-regulations included 42 patients. The ELF3/HNF4A up-regulated group showed a more advanced age (mean age: 68.4 years old) than the group without ELF3/HNF4A up-regulation (mean age: 59.2 years old, Student’s *t*-test *p* < 0.0001, Table 2). The group without ELF3/HNF4A up-regulation also had a higher percentage of early onset (diagnosis at age less than 50 years old) gastric cancers (21.4% vs. 4.3% in the group with ELF3/HNF4A up-regulation. Fisher’s exact test *p* = 0.02). No differences were observed between the groups in gender or tumor stage distribution (Table 2). In contrast significant differences between the groups were observed in tumor histology and tumor grade, with the group without ELF3/HNF4A up-regulation displaying more frequently diffuse histology (40.5% vs. 4.1% in the group with ELF3/HNF4A up-regulation, χ^2^ test *p* = 0.00003) and high grade (88.1% vs. 32.7% in the group with ELF3/HNF4A up-regulation, χ^2^ test *p* = 0.00001, Table 2).

**Table 2:** Clinical characteristics of the combined TCGA gastric cancer cohort with and without ELF3 and HNF4 over-expression and of the groups with over-expression of ELF3 (mRNA expression *z*-scores relative to normal samples >0.5) and over-expression of HNF4A (mRNA expression *z*-scores relative to normal samples >1) and without ELF3 (mRNA expression *z*-scores relative to normal samples <0.5) and HNF4A (mRNA expression *z*-scores relative to normal samples <0.5) over-expression. Other histologies include signet ring, papillary and mucinous. NA: Not available, NOS: Not otherwise specified

Characteristic	All (n = 91) (%)	ELF3 and HNF4A Over-Expressed (n = 49) (%)	ELF3 and HNF4A Not Over-Expressed (n = 42) (%)	*p*
**Age (mean)**	64.1 ± 11.1	68.4 ± 9.5	59.2 ± 10.8	<0.0001
**Early onset (≤50 years-old)**				
yes	11 (12.4)	2 (4.3)	9 (21.4)	0.02
no	78 (87.6)	45 (95.7)	33 (78.6)	
NA	2	2	0	
**Sex**				
Male	61 (67.0)	36 (73.5)	25 (59.5)	0.18
Female	30 (33.0)	13 (26.5)	17 (40.5)	
**Histology**				
Intestinal/NOS adenocarcinoma	65 (71.4)	44 (89.8)	20 (47.6)	0.00003
Diffuse	19 (20.9)	2 (4.1)	17 (40.5)	
Other	7 (7.7)	3 (6.1)	5 (11.9)	
**Grade**				
1–2	34 (37.4)	31 (63.2)	3 (7.1)	0.00001
3	53 (58.2)	16 (32.7)	37 (88.1)	
X	4 (4.4)	2 (4.1)	2 (4.8)	
**Stage**				
I	17 (18.9)	12 (24.5)	5 (12.2)	0.23
II	26 (28.9)	16 (32.7)	10 (24.4)	0.06 (i–ii vs. iii–iv)
III	37 (41.1)	17 (34.6)	20 (48.8)	
IV	10 (11.1)	4 (8.2)	6 (14.6)	
NA	1	0	1	

The genomic category of the tumors differed significantly in the 2 groups, with the group with ELF3/HNF4A up-regulation having mostly chromosomally unstable (CIN) tumors (80.4%), while in the group without ELF3/HNF4A up-regulation, about 40% of cases each were CIN and genomically stable (GS, Table 3). Only 10.5% and 15.25% of cases were MSI high in the 2 groups. The 2 groups did not differ also significantly in the percentage of cases with high (above 10/Mb) tumor mutation burden (TMB) (Table 2). However, 60% (6 of 10) of the cases with high TMB in the group with ELF3/HNF4A up-regulation and all 4 cases with high TMB in the group without ELF3/HNF4A up-regulation had TMB above 25 mutations/Mb. About 80% of cases in the group with ELF3/HNF4A up-regulation had an Aneuploidy score (AS) above 4% and 87% of cases had a Fraction of Genome Altered (FGA) index above 0.1, consistent with the high prevalence of CIN in the group (Table 3).

**Table 3:** Genomic characteristics of the combined TCGA gastric cancer cohort with and without ELF3 and HNF4 over-expression and of the groups with over-expression of ELF3 (mRNA expression *z*-scores relative to normal samples >0.5) and over-expression of HNF4A (mRNA expression *z*-scores relative to normal samples >1) and without ELF3 (mRNA expression *z*-scores relative to normal samples <0.5) and HNF4A (mRNA expression *z*-scores relative to normal samples <0.5) over-expression. CIN: Chromosomal Instability, MSI: Microsatellite Instability, GS: Genomically Stable, EBV: Epstein-Barr Virus, POLE: Polymerase epsilon, TMB: Tumor Mutation Burden, Mb: Megabase, AS: Aneuploidy Score, NA: Not available

Genomic characteristic	All (n = 91) (%)	ELF3 and HNF4A Over-Expressed (n = 49) (%)	ELF3 and HNF4A Not Over-Expressed (n = 42) (%)	*p*
**Genomic category**				
CIN	52 (61.9)	37 (80.4)	15 (39.5)	0.00009
GS	16 (19.0)	0	16 (42.1)	
MSI	11 (13.1)	7 (15.2)	4 (10.5)	
EBV	4 (4.8)	1 (2.2)	3 (7.9)	
POLE	1 (1.2)	1 (2.2)	0	
NA	7	3	4	
**TMB**				
High (>10 mutations/Mb)	14 (15.4)	10 (20.4)	4 (9.5)	0.24
Low (≤10 mutations/Mb)	77 (84.6)	39 (79.6)	38 (90.5)	
**AS**				
<4	29 (33.3)	10 (20.4)	19 (50.0)	0.01
4–24	54 (62.1)	36 (73.5)	18 (47.4)	
>24	4 (4.6)	3 (6.1)	1 (2.6)	
NA	4		4	
**FGA**				
<0.1	37 (40.7)	6 (12.2)	31 (73.8)	0.0001
>0.1	54 (59.3)	43 (87.8)	11 (26.2)	

**Figure 8:**
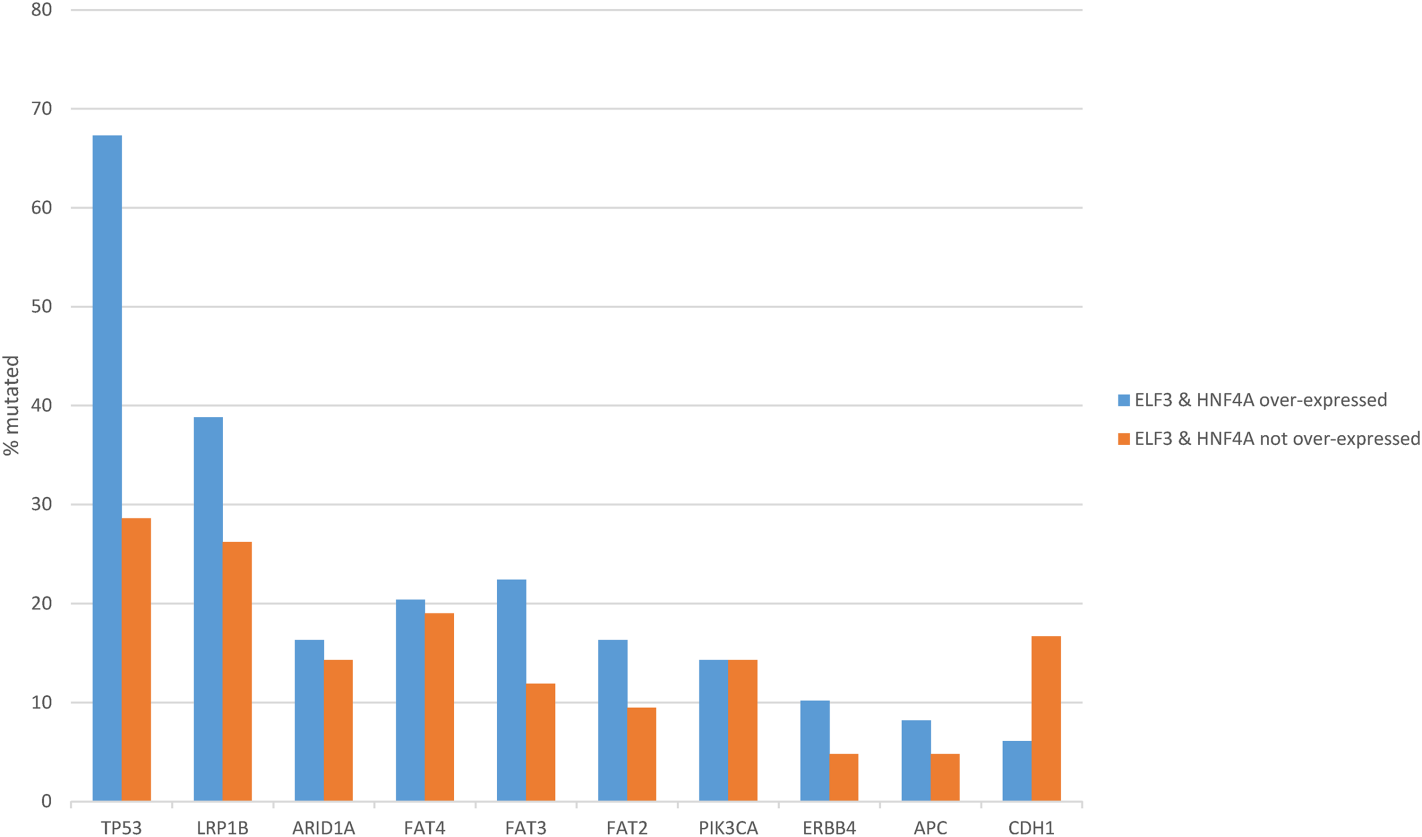
Prevalence of common gastric cancer-associated mutations in gastric cancer with ELF3 and HNF4A mRNA up-regulation vs. gastric cancers without ELF3 and HNF4A mRNA up-regulation. Data are from The Cancer Genome Atlas (TCGA)

Mutations in tumor suppressor *TP53*, the most frequently mutated cancer-associated gene in gastric cancers, were significantly more frequent in the ELF3/HNF4A up-regulated group (67.3%) compared with the non-up-regulated group (28.6%, Fisher’s exact test *p* = 0.0003, Fig. 8). Other less frequently mutated cancer-associated genes such as *LRP1B*, *FAT3*, *FAT2* and *ERBB4* were also more commonly mutated in the same group of patients with ELF3/HNF4A up-regulation, but differences did not reach statistical significance (Fig. 8). In contrast, *CDH1*, encoding for E cadherin, showed a higher mutation rate in ELF3/HNF4A non-up-regulated cancers (16.7% vs. 6.1% in the up-regulated group), which was not however statistically significant (Student’s *t*-test *p* = 0.17, Fig. 8).

**Figure 9:**
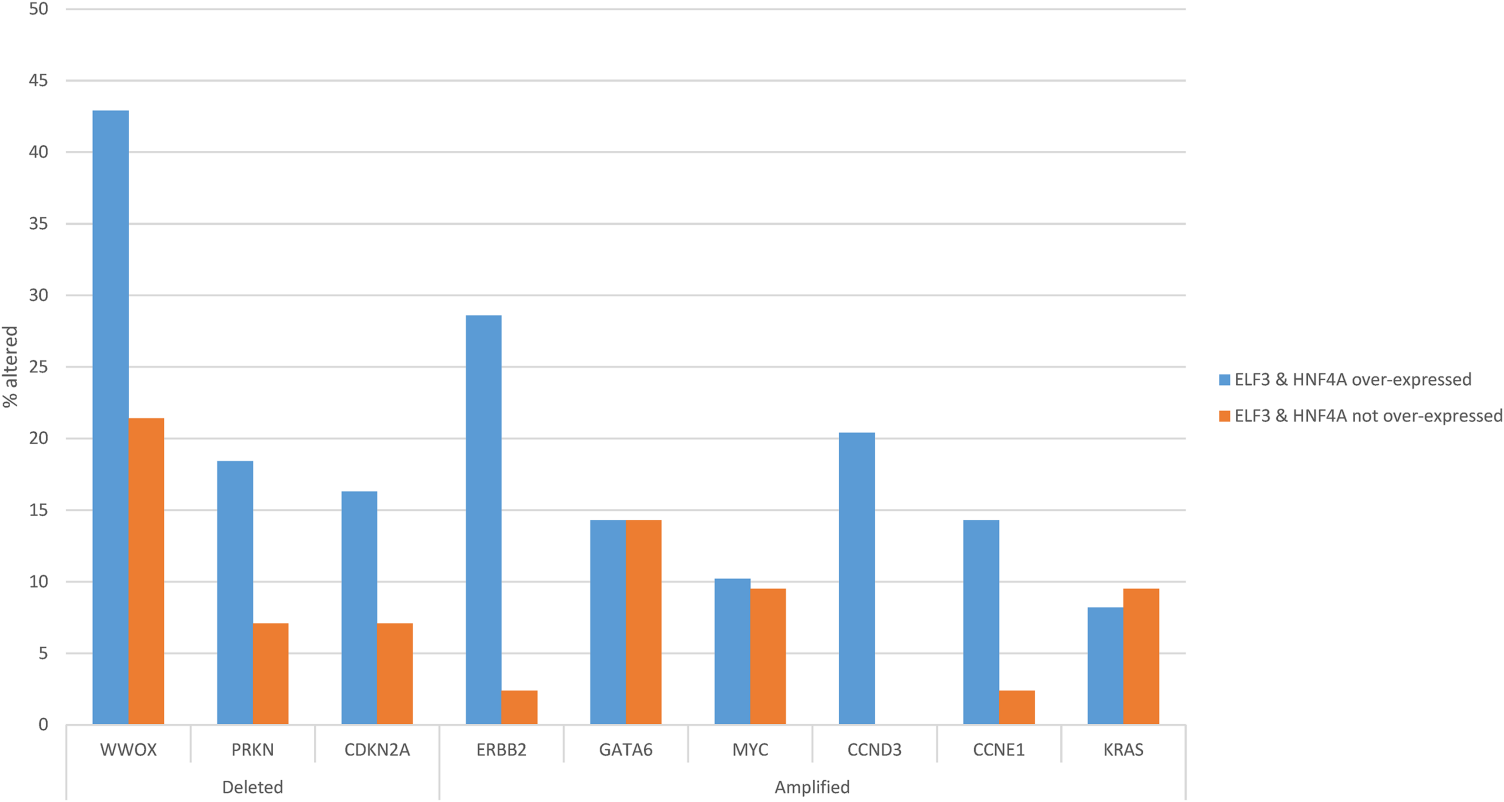
Prevalence of common gastric cancer-associated copy number alterations in gastric cancer with ELF3 and HNF4A mRNA up-regulation vs. gastric cancers without ELF3 and HNF4A mRNA up-regulation. Data are from The Cancer Genome Atlas (TCGA)

Regarding copy number alterations, the most commonly deleted locus in gastric adenocarcinomas at 16q23.1, a fragile site encoding for WW domain containing oxidoreductase WWOX, was twice as frequently deleted in ELF3/HNF4A up-regulated cancers (42.9%) compared with ELF3/HNF4A non-up-regulated gastric cancers (21.4%, Student’s *t*-test *p* = 0.04, Fig. 9). Other frequently deleted genes in gastric cancer, *PRKN* (encoding for ubiquitin ligase Parkin, also located at a fragile site), and for CDK inhibitor *CDKN2A*, were also more frequently observed in the up-regulated group, without reaching significance (Student’s *t*-test *p* = 0.13 and 0.21, respectively, Fig. 9). Amplifications of *ERBB2* and *CCND3* were significantly more frequent in ELF3/HNF4A up-regulated gastric cancers (28.6% and 20.4%, respectively) compared with the group of non-up-regulated cancers (2.4% and none, Student’s *t*-test *p* = 0.0006 and 0.001, respectively, Fig. 9). Another cyclin gene, *CCNE1* also showed a trend for higher amplification frequency in ELF3/HNF4A up-regulated cancers (Student’s *t*-test *p* = 0.06). Other frequently amplified genes in gastric cancer such as *MYC*, *GATA6* and *KRAS* displayed no differences between the two groups (Fig. 9).

**Figure 10:**
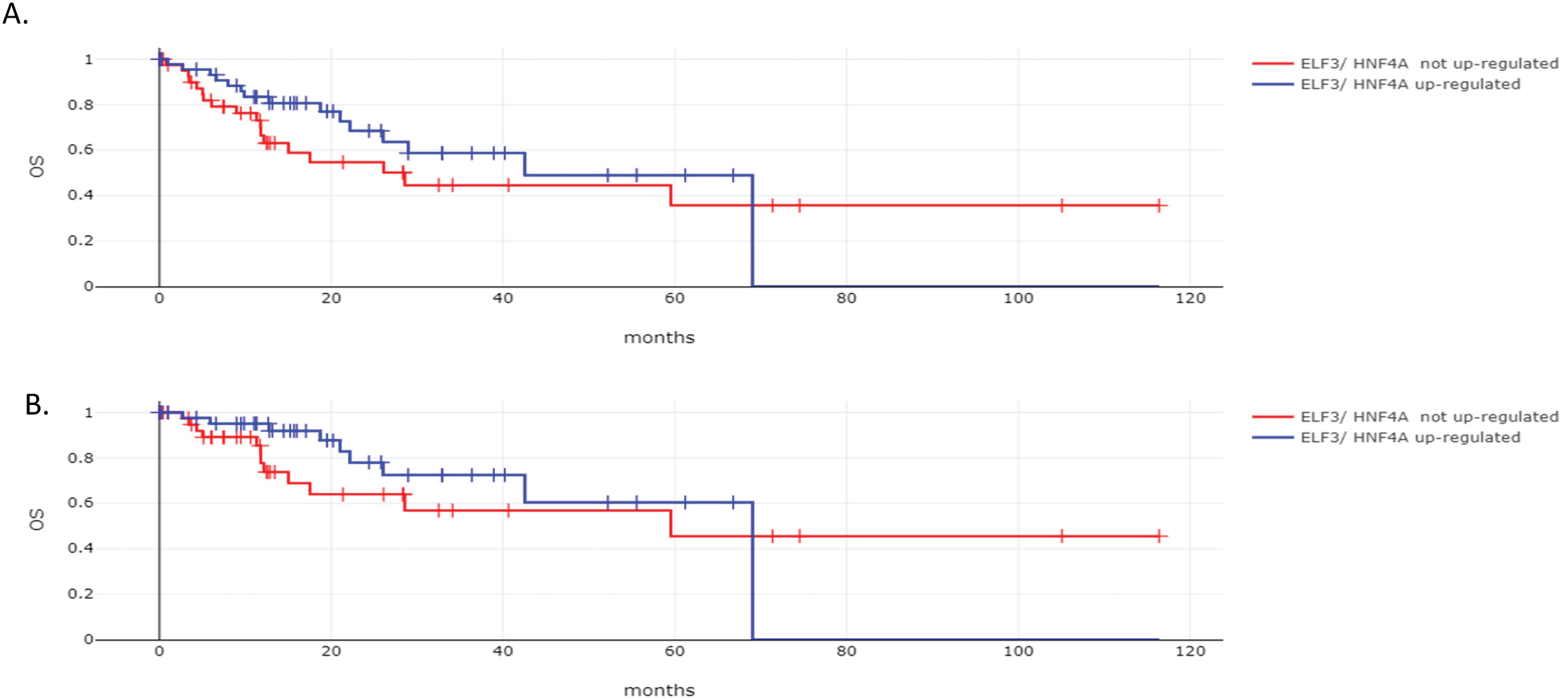
Survival outcomes according to ELF3 and HNF4A mRNA levels of expression. (**A**). Overall Survival (OS) and (**B**). Disease Specific Survival (DSS) of patients with gastric cancers with ELF3 and HNF4A mRNA up-regulation vs. gastric cancers without ELF3 and HNF4A mRNA up-regulation. Data are from The Cancer Genome Atlas (TCGA)

The overall survival (OS) of the two groups of gastric cancers with and without ELF3 and HNF4A concomitant up-regulation was not different (Log Rank test *p* = 0.24, Fig. 10A). The Disease Specific Survival (DSS) was also not different between the groups (Log Rank test *p* = 0.27, Fig. 10B).

## 4 Discussion

The role of ETS family transcription factors in gastric adenocarcinoma and their expression compared to normal gastric epithelia is of significant interest, given the involvement of this family of transcription factors in carcinogenesis. The ETS domain is the defining feature of the family and confers the DNA sequence binding specificity in target gene promoters. A few studies have examined specific family member factors of the family in gastric cancers. ETS1 was not expressed in normal gastric epithelium but was expressed in 51.6% of adenocarcinomas [22]. Higher ETS1 expression was associated with a more advanced stage and poorer survival [22]. ETS1, together with other transcription factors including AP-1, NF-κB, and HNF3β, has binding sites in the promoter of claudin 9 gene, and aberrant activity may lead to deregulation of tight junctions in gastric cancer [8]. PEA3 sub-family member ETV4 mRNA expression was observed in 64% of a series of 100 gastric cancer patients and was associated with adverse pathologic features including lymphatic and vascular invasion and lymph node positivity [23]. Tumors with positive ETV4 expression had worse progression-free survival (PFS) and OS. The ETS family member ERG cooperated with histone lysine methyltransferase SETDB1 to induce *CCND1* and matrix metalloproteinase 9 (*MMP9*) through promoter binding [24]. SETDB1 is induced by *H. pylori* infection through TCF4, suggesting a mechanism for this infection, which is associated with gastric cancer, to promote cell proliferation.

Another ETS family member, SAM, pointed domain containing ETS transcription factor (SPDEF) promotes the expression of transcription factor Forkhead Box M1 (FOXM1) by directly binding its promoter, which contains a core GGAT sequence, in gastric cancer cells [25]. FOXM1 reciprocally promoted expression of SPDEF as part of a feed-forward regulation loop.

In the overview of available genomic and proteomic data presented in the current report, a variable expression of ETS family transcription factors in gastric cancers was observed, with some transcription factors of the family being well expressed. These include ELF3, ETS2, EHF, ERF, and ELF1, while several other ETS family members are expressed at moderate levels. The rate of genomic alterations of individual members of the family in gastric cancer is low, but the rate of these alterations becomes substantial if they are considered as a group in specific genomic categories of gastric cancers. Up to 70% of MSI high gastric cancers, for example, possess one or more ETS family member mutations. Moreover, about one-third of CIN gastric cancers possess copy number alterations in one or more ETS family members. Although these alterations are currently of unknown pathogenic significance, they may contribute to the destabilization of crucial cancer-associated processes.

**Figure 11:**
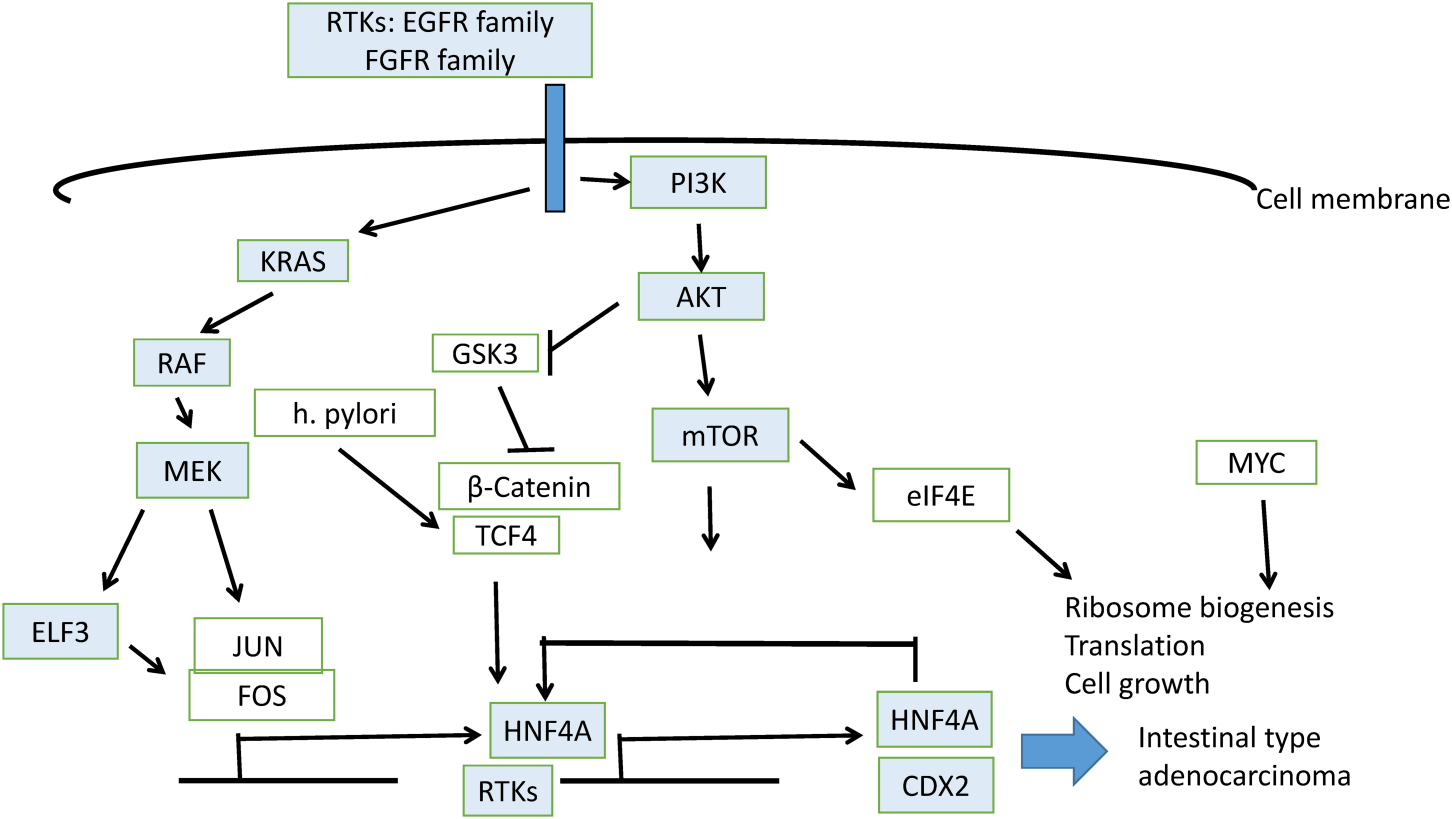
Schematic representation of pathways involved in gastric intestinal metaplasia induction leading to gastric carcinoma

ETS transcription factors are activated by receptor tyrosine kinase cascades, which induce their phosphorylation by kinase MEK (Fig. 11). Induction of expression of receptor tyrosine kinase genes, which possess ETS binding sites in their promoters, is part of feed-forward loops. For example, ELF3 induces HER2 in HER2-overexpressing cells [26]. A compound that disrupted the interaction of ELF3 with the Mediator subunit MED23 blocked expression of HER2 and inhibited trastuzumab-sensitive and resistant cells. In a study of gastric cancer patients, *ELF3* mutations together with mutations in the E cadherin gene, *CDH1*, and the polymeric immunoglobulin receptor *PIGR* gene were significantly more frequent in patients with peritoneal metastases compared with gastric cancer patients who did not develop metastases to the peritoneum [27]. In a cohort of 175 gastric cancer patients from China, *ELF3* was one of seven significantly mutated genes, together with *TP53*, *ARID1A*, *RHOA*, *RPL22*, *B2M*, and *OR6B1* [28]. In addition, ELF3, in combination with transcription factors KLF5 and GATA6, regulated the expression of another ETS family factor, EHF (ETS Homologous Factor), in gastroesophageal cancer cells [29]. Both ELF3 and EHF, together with ELF5, which is located on the same chromosome arm as EHF, at 11p13, belong to the ESE (Epithelial Specific ETS) sub-family and contain an amino-terminal pointed domain [30]. EHF, in conjunction with co-activator AJUBA, promoted signaling through the KRAS pathway. Activation of the KRAS/BRAF/ERK cascade may stem from the upregulation of receptor tyrosine kinase MET by EHF [31]. Other pathways activated downstream of MET include PI3K/PTEN/AKT, which results in inhibition of kinase GSK3, and the STAT3 pathway, which promotes epithelial to mesenchymal transition [31]. In EBV-associated gastric cancers in particular, EHF was up-regulated through a LMPA2 viral protein-mediated activation of STAT3, which resulted in enhanced cell growth [32]. ELF3, together with KLF5 and EHF overexpression, has been shown to characterize a subset of peritoneal metastatic gastric cancers through the induction of super-enhancers [33]. These data argue for a role of ELF3 in core transcription factor networks of gastric cancer pathogenesis, leading from dysplasia to intestinal metaplasia and cancer. In these networks, HNF4A is also a key participant and highly co-expressed with ELF3.

HNF4A is expressed widely in cancers of the gastrointestinal tract, biliary tree, and pancreas as opposed to HNF6, encoded by the *ONECUT1* gene, whose expression is restricted to liver and small bile duct carcinomas [34]. In hepatocyte-like cells from hepatic organoids, HNF4A was expressed in conjunction with ELF3, and the expression of the latter decreased hepatocyte differentiation of these organoids compared with primary human hepatocytes, which did not express ELF3 [35]. ELF3 depletion led to the induction of liver markers in organoid cultures. In gastric cancer, HNF4A expression is stronger than that in the adjacent unaffected mucosa [36]. ELF3, together with other master transcription factor regulators of the gastrointestinal epithelium, which include GATA4, GATA6, and KLF5, induces HNF4A, which then contributes to the up-regulation of its own expression [11] (Fig. 11). HNF4A activates CDX2 in gastric intestinal metaplasia, which is a precursor of intestinal adenocarcinoma [37].

High co-expression of ELF3 and HNF4A is shown in the current study to lead predominantly to gastric cancers with intestinal histology, lower grade, commonly earlier stage, and the CIN phenotype compared with cancers without up-regulated ELF3/HNF4A co-expression. As expected by the high frequency of CIN, the ELF3/HNF4A up-regulated group had higher rates of common gastric cancer amplifications and deletions than the group without ELF3/HNF4A up-regulation, and, in addition, it displayed a high rate of *TP53* mutations. As these mutations are associated with treatment resistance, their presence may counteract the lower risk clinicopathologic features associated with ELF3/HNF4A up-regulated cancers and could explain the lack of statistically significant differences in survival outcomes [38]. However, this may also relate to the low number of patients in the two groups. A similar increased rate of *TP53* mutations has been described in gastric cancers with up-regulation of CDX2 [12]. The absence of ELF3/HNF4A up-regulation seems to define a subgroup of gastric cancers with high rates of genomic stability and diffuse histology. Therefore, the two transcription factors may underline fundamental differences in gastric carcinogenesis pathways, with associations to intestinal metaplasia or the loss of adhesions/epithelial-mesenchymal transition pathway. In this regard, the coordinated expression of ELF3 and HNF4A with other factors promoting intestinal metaplasia, such as CDX2, is noteworthy and could provide leads for therapeutic or prevention measures, as CDX2 induction has been described as a direct result of *Helicobacter pylori* infection or bile acid-related metaplasia [39]. These physical insults in the gastric environment act at the cellular level on a chronic basis to trigger pro-carcinogenic processes [40]. The molecular pathway from *h. Helicobacter pylori* infection to intestinal metaplasia, dysplasia, and cancer in the epithelium of the stomach has been described as a linear process, although in some cases and depending on the instigating agent, some stages of the cascade can be bypassed [41–43]. Metaplasia-associated transcription factors are up-regulated during the process and, although initially changes are reversible with eradication of the insult such as *h. Helicobacter pylori*, later, epigenetic changes lead to genomic changes such as mutations that produce irreversible lesions, such as advanced dysplasia and cancer, which are not dependent on ongoing infection [42].

ELF3 acts as an oncogene or a tumor suppressor in different contexts [44]. Despite being over-expressed in a sub-set of gastric cancers, ELF3 has functions that are generally associated with tumor suppression such as repressing epithelial-mesenchymal transition (EMT) and promoting the reverse process, mesenchymal to epithelial transition (MET) [45,46]. The two processes, EMT and MET are hijacked by cancer cells from embryogenesis, where they contribute to organogenesis and fetal development [47,48]. In cancer, EMT is involved in cell invasion and initiation of metastasis, and MET is involved in the establishment of metastatic cells in remote sites [49]. Therefore, MET can contribute to cancer dissemination if it occurs in metastatic sites. In contrast, at the primary tumor location, before the complete EMT has taken place, dissociating cancer cells from the neighboring cells, the MET network can counteract EMT forces, and, in this case, MET and associated factors could be tumor suppressive. ELF3 has been recognized as essential in MET and is associated with the establishment of E-cadherin in the plasma membrane during the process [46]. In pre-cancerous gastric epithelia, the network promoting intestinal metaplasia, centered on CDX2, HNF4A, GATA4, and ELF3, represents an attempt at adaptation to chronic inflammation, such as due to *H. pylori*. This adaptation is successful if complete metaplasia (also called small intestinal type) is achieved, but can be pro-tumorigenic if the process progresses to incomplete (colonic type) metaplasia [50,51]. In that case, metaplastic cells have the highest risk of further progress to dysplasia and cancer [52]. Along the process of transformation and after cancer development, the level of expression of the factors of the network may vary in a non-linear manner and promote different histologies with higher or lower differentiation, as observed in the current study. Therapeutically, these observations further support an individualized approach based on biomarkers depicting the status of the network, before attempting to disrupt it. Detailed dissection of the role of ELF3 and HNF4A in gastric cancer could be performed in studies using CRISPR or siRNA models.

Direct targeting ELF3 therapeutically with small molecule inhibitors is currently not feasible in the clinic, as there are no clinically grade chemical probes available. Transcription factors have not been easily targetable as they lack enzymatic activity and instead function as adaptor proteins to bring the transcription machinery to specific DNA sequences and promote initiation of transcription of target genes [53]. Nevertheless, a transcription factor of the nuclear transcription factor super-family, Estrogen Receptor (ER), was one of the first specific therapeutic targets in oncology, inhibited by tamoxifen. This targeting took advantage of the need for a ligand for activation of the ER transcription function, estradiol, with an associated presence of a ligand binding pocket in the tertiary structure of ER, appropriate for an inhibitor binding. Transcription factors without known ligands, such as ELF3, could be targeted, instead, by drugs that interfere with their interaction with the DNA or the general transcription machinery. As mentioned above, an inhibitor of the interaction of ELF3 with the component of Mediator, MED23, has been published and is commercially available for research [54]. This inhibitor, called YK1, was shown to be effective in colorectal cancer models with activation of the HER2/KRAS pathway due to oncogenic *KRAS* G12D mutations [54]. Alternatively, ELF3 could be marked for degradation by Proteolysis Targeting Chimeras (PROTACs), which are constructs associating a receptor binding a target protein with a ubiquitin ligase, promoting the ubiquitination and subsequent proteasome degradation of the target protein [55]. However, the creation of an ELF3 targeting PROTAC would require the discovery of a probe that would bind it. The clinical feasibility of the PROTAC concept has been validated in the case of ER, with an ER-targeting PROTAC, vepdegestrant, being in advanced phases of clinical development [56]. In addition, both a small molecule inhibitor, JQ1, and an associated PROTAC using JQ1 as a target ligand have been discovered and tested for another protein involved in transcription, BRD4 (Bromodomain containing protein 4), which bridges transcription factors and co-factors with the general transcriptional machinery [57,58]. These early successes in inhibiting BRD4 not only serve as a proof of concept for the inhibition of proteins involved in transcription but can also have direct relevance in down-regulation of lineage-defining factors and networks of transcription factors, such as ELF3 in gastric cancer, which depend more heavily on BRD4 and super-enhancer-mediated transcription [59]. ELF3 expression was confirmed to be dependent on the function of an upstream super-enhancer in villous trophoblast cells [60]. Interestingly, the super-enhancer was dependent on the inhibition not only of BRD4, but also of ELF3 itself, suggesting a positive feedback loop, in which ELF3 regulates its own expression [60]. In a murine esophageal adenocarcinoma model, the ELF3 super-enhancer was up-regulated by nuclear receptor transcription factor peroxisome proliferator activating receptor γ (PPARγ) which is induced by a high-fat diet [61]. However, as discussed also in the previous paragraph, caution is needed in inhibiting ELF3, as the transcription factor may act as a tumor suppressor in certain contexts. Salient to this, in certain cell types, such as synovial fibroblasts, ELF3 may have pro-inflammatory functions through the induction of cytokines [62]. Chronic tissue inflammation is associated with carcinogenesis, while in established cancers, it may promote an anti-tumor immune response. Still, in other cell types, such as lung carcinoma cells with activation of receptor tyrosine kinase pathways due to mutations in EGFR or KRAS, ELF3 had a clear tumor-promoting effect, and blocking its function through knockdown or inhibition of its upstream inducer, kinase Protein Kinase C iota (PKCι), resulted in cell apoptosis [63].

In the limitations of this investigation, it should be acknowledged that the study relies on secondary data available in the public domain and not on new experiments or observations, and that it examines one cohort. However, the source data were multi-institutional and extensive. The associations of expression of core gastric intestinal metaplasia transcription factors described in this study can be the result of direct regulation, as it has been reported for factor CDX2, but could alternatively be indirect, through other factors. The current study, by its observational design, cannot dissect mechanistic relationships. Other limitations include a limited cohort size for the main comparison between groups with or without ELF3/HNF4A over-expression, potential biases in the publicly available source datasets, and a lack of information on putative confounding factors such as treatment history or patient ethnic background.

In conclusion, the pathogenesis of gastric cancer is related to chronic insults affecting a network of transcription factors, including members of the ETS family. ELF3 is a prominent member with relevance in gastric cancer, and its up-regulation, observed in sub-sets of the disease, defines cancers with specific characteristics that may provide targeted therapeutic opportunities. The high level of CIN in gastric cancers with up-regulation of ELF3 and HNF4A is a feature not currently clinically targetable, but efforts to develop drugs are ongoing. An example is inhibitors of kinase TTK1 (Threonine Tyrosine kinase 1), which has important functions during the mitotic spindle assembly checkpoint, and its inhibition would accentuate CIN and chromosome missegregation [64]. Development of such drugs would greatly benefit from the identification of genomic subsets of gastric cancer based on biomarkers of sensitivity [65,66]. Efforts for direct targeting of ELF3 are also pursued, with reports on interference with the general transcriptional machinery being successful in preclinical models. Recent advances in artificial intelligence-aided protein structure resolution and prediction of interactions with other biologic macromolecules and small molecules, such as drugs, will certainly accelerate the discovery of inhibitors for ELF3 [67]. The power of the recently published Alphafold3 algorithm has been hailed as a game-changer in this respect. It is hoped that by exploiting these new tools, candidate drugs will be quickly discovered and could become swiftly optimized in the preclinical phase, accelerating their clinical introduction.

## Data Availability

All data generated for this study are included in the manuscript and no additional data are available.
